# Femtosecond gas-phase mega-electron-volt ultrafast electron diffraction

**DOI:** 10.1063/1.5120864

**Published:** 2019-10-15

**Authors:** X. Shen, J. P. F. Nunes, J. Yang, R. K. Jobe, R. K. Li, Ming-Fu Lin, B. Moore, M. Niebuhr, S. P. Weathersby, T. J. A. Wolf, C. Yoneda, Markus Guehr, Martin Centurion, X. J. Wang

**Affiliations:** 1 SLAC National Accelerator Laboratory, 2575 Sand Hill Road, Menlo Park, California 94025, USA; 2 Department of Physics and Astronomy, University of Nebraska-Lincoln, Lincoln, Nebraska 68588, USA; 3 Stanford PULSE Institute, SLAC National Accelerator Laboratory, Menlo Park, California 94025, USA; 4 Linac Coherent Light Source, SLAC National Accelerator Laboratory, Menlo Park, California 94025, USA; 5 Institut für Physik und Astronomie, Universität Potsdam, 14476 Potsdam, Germany

## Abstract

The development of ultrafast gas electron diffraction with nonrelativistic electrons has enabled the determination of molecular structures with atomic spatial resolution. It has, however, been challenging to break the picosecond temporal resolution barrier and achieve the goal that has long been envisioned—making space- and-time resolved molecular movies of chemical reaction in the gas-phase. Recently, an ultrafast electron diffraction (UED) apparatus using mega-electron-volt (MeV) electrons was developed at the SLAC National Accelerator Laboratory for imaging ultrafast structural dynamics of molecules in the gas phase. The SLAC gas-phase MeV UED has achieved 65 fs root mean square temporal resolution, 0.63 Å spatial resolution, and 0.22 Å^−1^ reciprocal-space resolution. Such high spatial-temporal resolution has enabled the capturing of real-time molecular movies of fundamental photochemical mechanisms, such as chemical bond breaking, ring opening, and a nuclear wave packet crossing a conical intersection. In this paper, the design that enables the high spatial-temporal resolution of the SLAC gas phase MeV UED is presented. The compact design of the differential pump section of the SLAC gas phase MeV UED realized five orders-of-magnitude vacuum isolation between the electron source and gas sample chamber. The spatial resolution, temporal resolution, and long-term stability of the apparatus are systematically characterized.

## I. INTRODUCTION

The study of the photoinduced dynamics of small isolated molecules is of crucial importance to the understanding of structure-function relationships in nature.^1,2^ Given adequate temporal and spatial resolutions, the imaging of structural changes following photoexcitation can provide a glimpse into the mechanisms governing the conversion of light into chemical and mechanical energy. Ultrafast diffraction techniques use probes with a short wavelength (≤1 Å) and short pulse duration (≤100 fs) to access structural information on the relevant spatial and temporal scales. The most powerful probes for ultrafast diffraction are X-rays and electrons.^3,4^ The advent of X-ray free-electron lasers (XFELs)^5–7^ has enabled time-resolved X-ray diffraction of gas-phase targets with Angstrom-level spatial resolution and sub-100 fs temporal resolution.^8–10^ Electron diffraction has been shown to be well-suited to the study of structural dynamics in the gas phase with sub-Å spatial resolution because of the large scattering cross section and the short de Broglie wavelength of the electrons.^11^ Since the first reported gas electron diffraction (GED) experiments, GED has become a standard technique for determination of the time-averaged structures of molecules in the gas phase.^12,13^ By implementing pulsed electron sources, time-resolved GED was able to structurally resolve the nanosecond dynamics of long-lived photofragments.^14,15^ The advent of commercial femtosecond lasers and developments in pulsed photo-electron sources allowed the pioneering work of Zewail and Weber to bring GED into the picosecond domain, establishing the field of ultrafast gas electron diffraction (UGED). Despite the remarkable scientific impact of its contribution on the study of transient structures,^16,17^ early UGED experiments were unable to achieve subpicosecond temporal resolution.^18–23^ However, a resolution in the order of 100 fs or even less is needed to capture relevant nuclear motion in many photoinduced processes. Reaching ≤100 fs time resolution using electrons at kilo-electron-volt (keV) energies remains a challenge, even in the modern UGED apparatus.

The temporal resolution, or instrument response function, of a UGED experiment, in which a gas sample is excited (pumped) by a laser pulse and probed by an electron bunch, is given by

$$\tau =\sqrt{\tau_{L}^{2}+\tau_{e}^{2}+\tau_{VM}^{2}+\tau_{\mathrm{VOA}}^{2}},$$ (1)

where τ_L_ is the pump laser pulse duration, τ_e_ is the probe electron bunch length, τ_VM_ is the velocity mismatch between the laser pulse and the electron bunch,^24^ and τ_TOA_ is the time-of-arrival (TOA) jitter between the pump laser and the probe electron bunch. In most cases, the temporal resolution of UGED is not limited by the pump pulse, τ_L_, since sub-50 fs laser systems are commercially available. The electron bunch length, τ_e_, is determined by the initial energy spread in the electron bunch and the space-charge induced pulse broadening during propagation, which often results in a temporal resolution of several hundred femtoseconds. Temporal resolution in the order of hundred femtoseconds has been demonstrated in keV UED experiments on solid-state samples using compact direct-current electron guns with the distance between the gun cathode and the sample in the order of 1 cm and a limited number of electrons per bunch.^25–27^ However, the use of compact electron guns in UGED is technically difficult as electron sources require an ultrahigh vacuum (≤10^−10 ^Torr) to avoid the electrical breakdown, while the pressure at the gas-sample chamber can easily exceed 10^−5 ^ Torr.

An alternative approach to obtain short electron bunches involves the use of a radio frequency (RF) cavity to temporally compress the electron bunch at the sample.^28,29^ Time-stamping^30^ and a sophisticated laser-rf synchronization^31^ have been used to minimize the TOA jitter associated with RF compression. Some keV UGED apparatus fitted with RF compression have demonstrated ∼500 fs temporal resolution.^32^ The temporal resolution of UGED experiments using keV (subrelativistic) electrons is dominated by the velocity mismatch between the pump laser and the probe electron, τ_VM_, associated with a typical gas target thickness much larger than a few micrometers. For example, using a 100-keV electron beam traversing a 200-*μ*m gas jet collinearly to the laser pump beam, the velocity mismatch contribution to the temporal resolution is found to be 550 fs. To circumvent this effect, laser pulse-front tilting can be used to compensate for the pump-probe velocity mismatch with limited sucess.^32–34^

The use of mega-electron-volt (MeV) high-brightness electron beams has been shown to significantly improve the temporal resolution of UGED.^35,36^ Space charge forces scale as 1/(β^2^γ^3^), where β=v/c, v is the velocity of the charged particle, c is the speed of light, and $\gamma =1/\sqrt{1-\beta^{2}}$. As a result, the space-charge repulsion of an electron beam with 3.7-MeV kinetic energy is a thousand times smaller than that of its 100-keV counterpart. In UGED apparatus using MeV electrons, the sample chamber can be moved away from the electron gun without severely compromising the electron bunch length. This added separation between the electron source and the target allow the implementation of more effective differential pumping designs, discussed in Sec. II. Moreover, the relativistic nature of MeV electrons almost eliminates the velocity mismatch. For example, in the case of a 3.7-MeV electron bunch traversing a 200-*μ*m gas sample colinearly to a laser beam, τ_VM_ is less than 10 fs.

Decades of intensive research and development efforts have been devoted to improving the performance of MeV electron sources,^37–47^ which has paved the way to the implementation of MeV ultrafast electron diffraction (MeV UED) for imaging ultrafast structural dynamics of molecules in the gas phase.^48^ In this paper, we report on the experimental demonstration of the SLAC gas-phase MeV UED apparatus capable of 70 fs root mean square (rms), or 150 fs full-width-at-half-maximum (FWHM), temporal resolution (instrument response function), 0.63 Å spatial resolution, and 0.22 Å^−1^ reciprocal-space resolution, which has enabled molecular movies, capturing the rotational dynamics in N_2_,^49^ vibrational dynamics in I_2_,^50^ a nuclear wave packet crossing a conical intersection in CF_3_I,^51^ and ring-opening in 1,3-Cyclohexadiene (CHD).^52^ The high resolution and sensitivity of the SLAC gas-phase MeV UED apparatus has also enabled the identification and study of ultrafast events with onsets separated by 70 fs.^51,52^ In this paper, we present the SLAC gas-phase MeV UED apparatus, its design layout, resolution, and stability characterization.

## II. EXPERIMENTAL SETUP

**FIG. 1.**
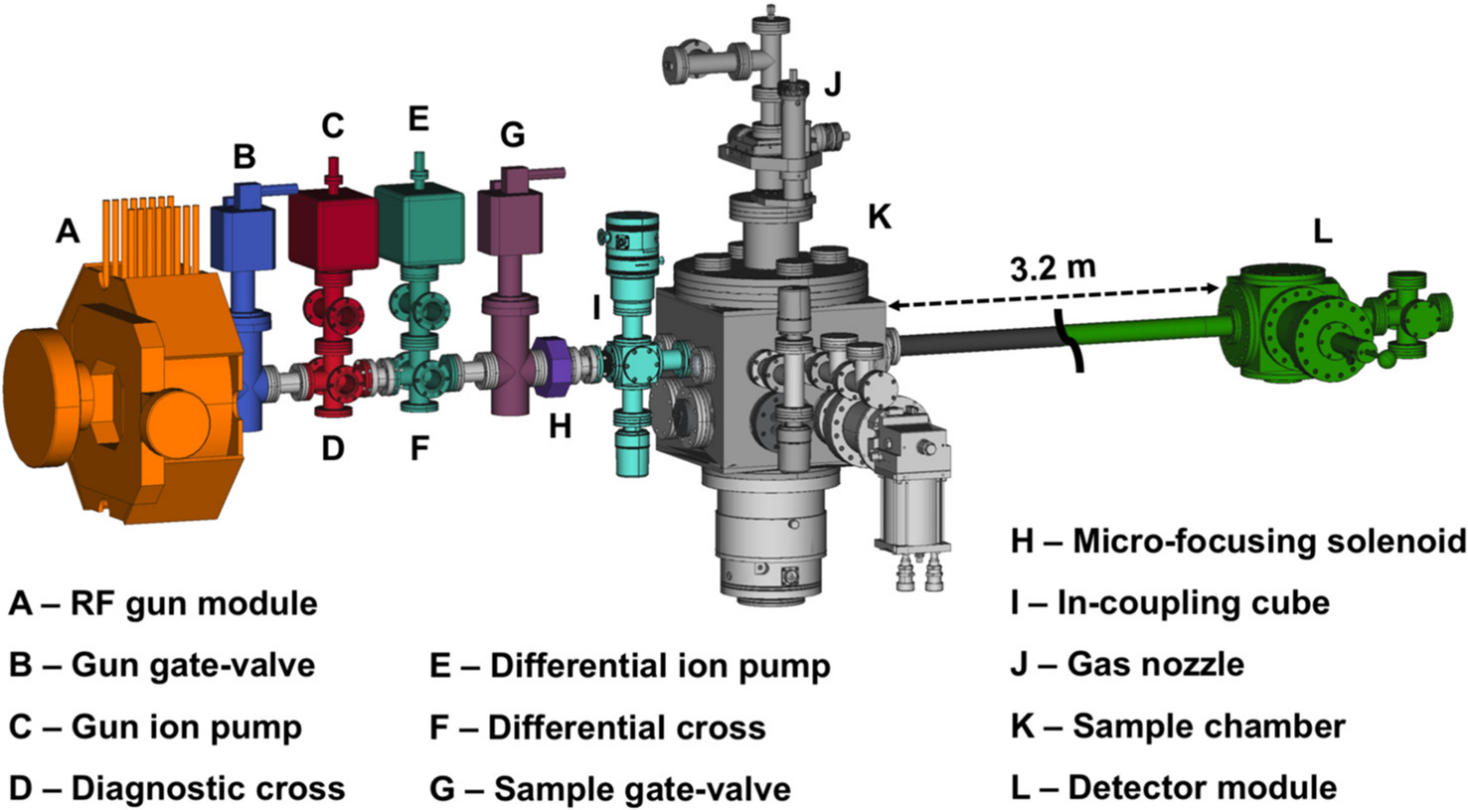
Schematic of the gas-phase MeV UED at SLAC.

**FIG. 2.**
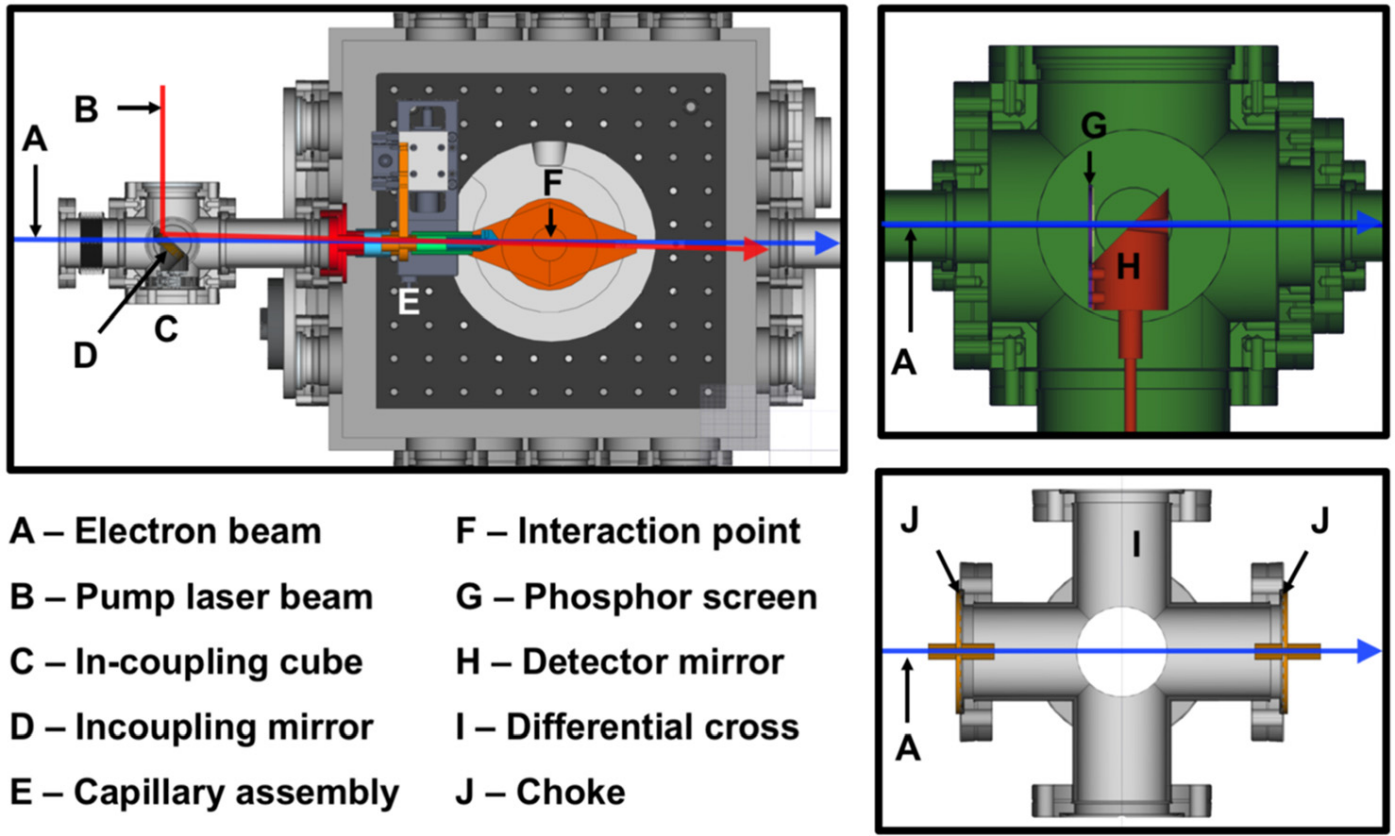
Cross-sectional view of the gas chamber (top left), the invacuum electron detector module (top right), and the differential pumping section (bottom right).

**FIG. 3.**
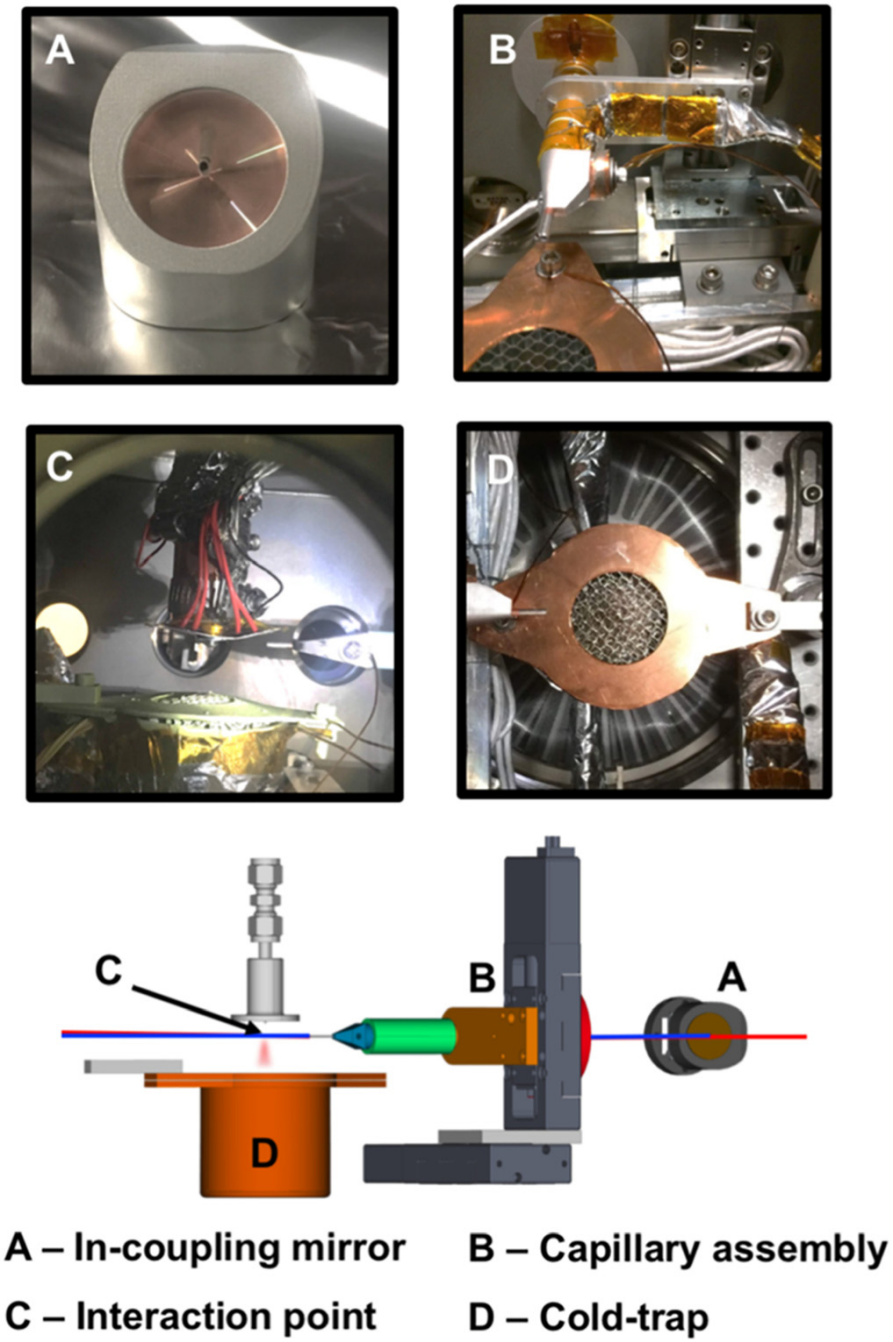
Photographs and computer-aided design (CAD) drawings of the incoupling mirror, capillary assembly, interaction point, and cold-trap.

The gas-phase MeV UED apparatus, schematically depicted in Fig. 1, has been developed around the preexisting MeV UED beamline housed in the Accelerator Structure Test Area (ASTA) at SLAC and described elsewhere.^46,47^ The apparatus employs an S-band 1.6-cell photocathode RF gun to produce high-brightness 3.7-MeV electron beams.^53^ Downstream of the electron gun, a diagnostic cross containing a beam profile monitor, movable Faraday cup, and motorized collimator made of Tungsten with fixed-size apertures (100, 200, and 500-*μ*m diameter), allows accurate manipulation and measurement of the beam parameters. Following a series of differential pumping sections (a sectional view shown in the bottom right panel of Fig. 2), a microfocusing solenoid provides additional control over the electron beam focusing.^47^ A third differential pumping section, housing the pump laser incoupling optics and fitted with a 30 L/s turbo pump, ensures adequate vacuum isolation between the RF gun and the sample chamber. In the case of sudden pressure spikes, two gate-valves with vacuum interlocks protect the electron gun from contamination. A movable capillary (2 mm inner diameter), protruding into the sample chamber, ensures two orders-of-magnitude pressure difference between the incoupling mirror and the sample chamber, thus preventing the contamination of the mirror surface by gas molecules. A schematic representation of the incoupling mirror and differential capillary assembly is shown in Fig. 3. Inside the sample chamber, a 3-dimensional translation stage ensures accurate positioning of the gas nozzle with respect to the pump-probe overlap region, referred to as the interaction point. Samples can be delivered to the interaction point using either a heated pulsed nozzle or a continuous flow cell, depending on the sample properties. Immediately underneath the interaction point, a cold trap consisting of a series of staggered honeycomb structures is cryocooled to 70 K to condense the exhausted sample, as shown in Fig. 3. A vertically mounted 1000 l/s turbo pump maintains the chamber vacuum below 5 × 10^−5 ^Torr, while the three differential pumping stages upstream ensure 5 orders-of-magnitude vacuum isolation, keeping the RF gun vacuum at ≤4 × 10^−10 ^Torr. The electron detector system is located 3.2-m downstream of the sample chamber. The detector module, shown in the top right panel of Fig. 2, is composed of a 4-cm-diameter phosphor screen positioned perpendicular to the electron beam path and a high-reflectivity mirror oriented 45° with respect to the phosphor screen. The phosphor screen is imaged through a vacuum viewport onto an Andor iXon Ultra 888 electron-multiplying charge-coupled device (EMCCD) camera using the mirror and an out-of-vacuum 40-mm f/0.85 lens.^54^ To prevent sensor saturation, a 2.9-mm-diameter hole at the center of the phosphor screen allows the passage of the undiffracted electron beam with orders-of-magnitude higher beam charge compared to the diffracted beams. Correspondingly, a hole at the center of the mirror allows the undiffracted electron beam to pass to a downstream beam dump. The shape of this hole was carefully designed to minimize the background associated with stray pump laser light. While a uniform hole allows stray pump laser light going through the phosphor hole to reflect and scatter within the hole and eventual hit the back of the phosphor, contributing to the diffraction pattern background; the smooth tapered hole design here implemented, has been found to eliminate these reflections and thus significantly reduce the pump-laser-induced background signal.

**FIG. 4.**
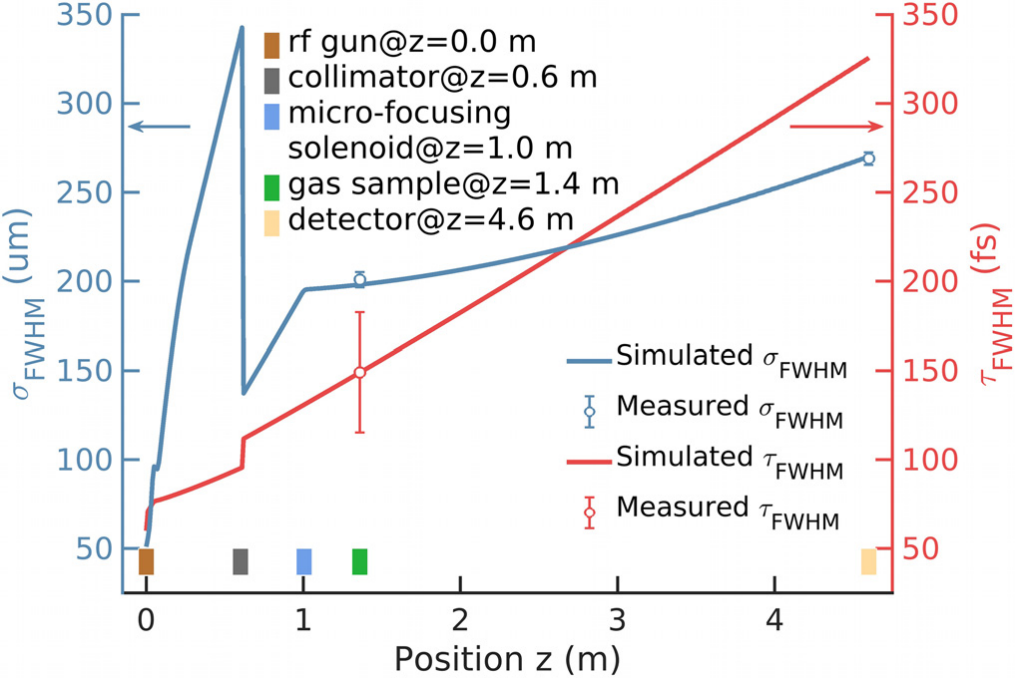
Comparison of the simulated and measured electron beam spot size, σ_FWHM_, and the bunch length, τ_FWHM_. The rectangles mark the positions of different apparatus components.

The gas-phase MeV UED apparatus is driven by a Ti:Sapphire laser system, producing 25-mJ, 50-fs laser pulses with a central wavelength of 800 nm at the repetition rate of 180 Hz. This laser is split into two parts: a 0.8-mJ branch is frequency tripled to 266 nm for electron generation at the RF gun photocathode, while the remainder in a second branch is reserved for the generation of pump wavelengths of 400 and 266 nm through harmonic generation using β-barium borate crystals, as well as tunable wavelengths in the visible and ultraviolet range using an optical parametric amplifier and frequency mixing. A low-level RF-laser timing system and a high stability RF power source regulate the rms pump-probe timing jitter to <50 fs.^46^ In the RF gun, electron pulses with 10 fC bunch charge at 3.7 MeV kinetic energy are generated; a circular collimator with 200-*μ*m diameter transmits the core portion of the beam, with a reduced charge of 2 fC; finally, the microfocusing solenoid focuses the electron beam to a 270-*μ*m FWHM spot size at the detector, which corresponds to 0.22 Å^−1^ reciprocal-space resolution with the underlying diffraction geometry. At the interaction point, the FWHM electron bunch length is estimated to be 150 fs (upper limit), and the FHWM beam size is measured as 200 *μ*m. These results, which will be described in Sec. III, are in good agreement with beam dynamics simulations carried out using General Particle Tracer,^55^ as shown in Fig. 4. Typical machine parameters are summarized in Table I.

**TABLE I.** Typical machine parameters for the gas-phase MeV UED apparatus.

Parameters	Values
Repetition rate	180 Hz
Vacuum at the rf gun	$\leq 4\times 10^{-10}$ Torr
Vacuum at the sample chamber	$\leq 5\times 10^{-5}$ Torr
Electron beam kinetic energy	3.7 MeV
Electron beam charge from the rf gun	10 fC
Collimator (z = 0.6 m) diameter	200 *μ*m
At the gas target (z = 1.4 m)	
Collimated electron beam charge	2 fC
Electron bunch length (FWHM)	<150 fs
Electron beam size (FWHM)	200 *μ*m
Pump laser spot size (FWHM)	300 *μ*m
Gas sample size (FWHM)	300 *μ*m
Electron beam transverse pointing jitter (rms)	<14 *μ*m
Laser-electron time-zero fluctuation (rms)	21.5 fs
At the electron detector (z = 4.6 m)	
Electron beam size (FWHM)	270 *μ*m
Reciprocal space resolution	0.22 Å^–1^
Spatial resolution	0.63 Å

**FIG. 5.**
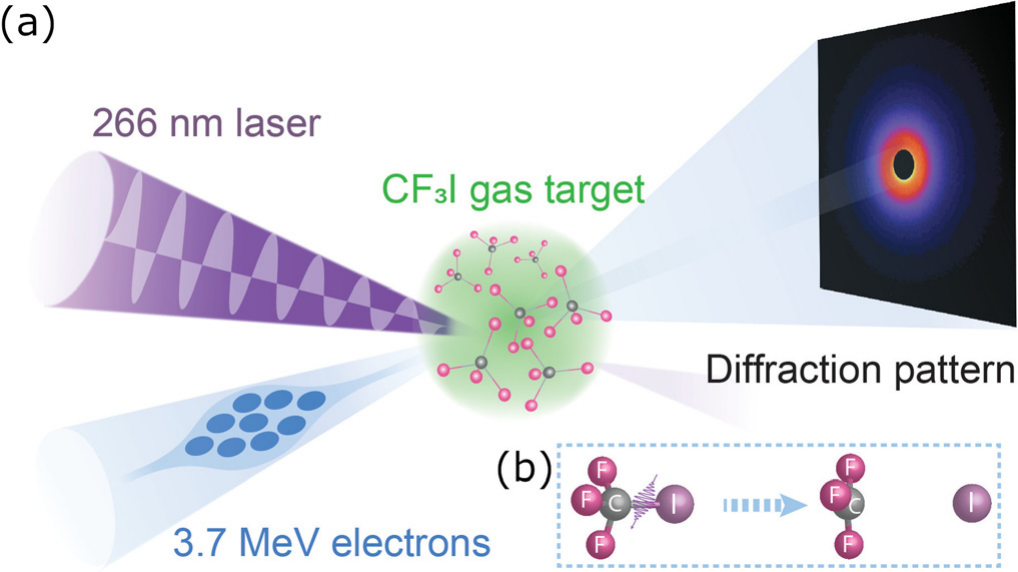
(a) Schematic of the interaction region in the gas-phase MeV UED sample chamber. Gas-phase CF_3_I molecules from the gas nozzle form pulsed gas jet samples (green). An ultrafast 266 nm pump laser (purple) excites the CF_3_I molecules, while a 3.7 MeV electron pulse (blue) probes the ultrafast structural dynamics from the excited CF_3_I molecules. Diffraction patterns as a function of the time delay between the pump laser and the probe electron pulses are captured on the electron detector 3.2 m downstream of the interaction point. (b) A cartoon of the CF_3_I photodissociation process.

The experimental conditions and interaction point geometry of a typical gas-phase MeV UED experiment are exemplified by the study of the photodissociation dynamics of trifluoroiodomethane (CF_3_I)^51^ illustrated in Fig. 5, where 266-nm pump laser pulses and 3.7-MeV electron probe bunches copropagate with <2° angle through a gas jet with a diameter of 300 *μ*m. A motorized delay stage installed in the pump laser optical path controls the relative time delay between the pump laser and the probe electron pulses. Diffraction patterns at different pump-probe delays were acquired at the detector with momentum transfer up to 12 Å^−1^.

## III. APPARATUS RESOLUTION CHARACTERIZATION

### A. Temporal resolution

**FIG. 6.**
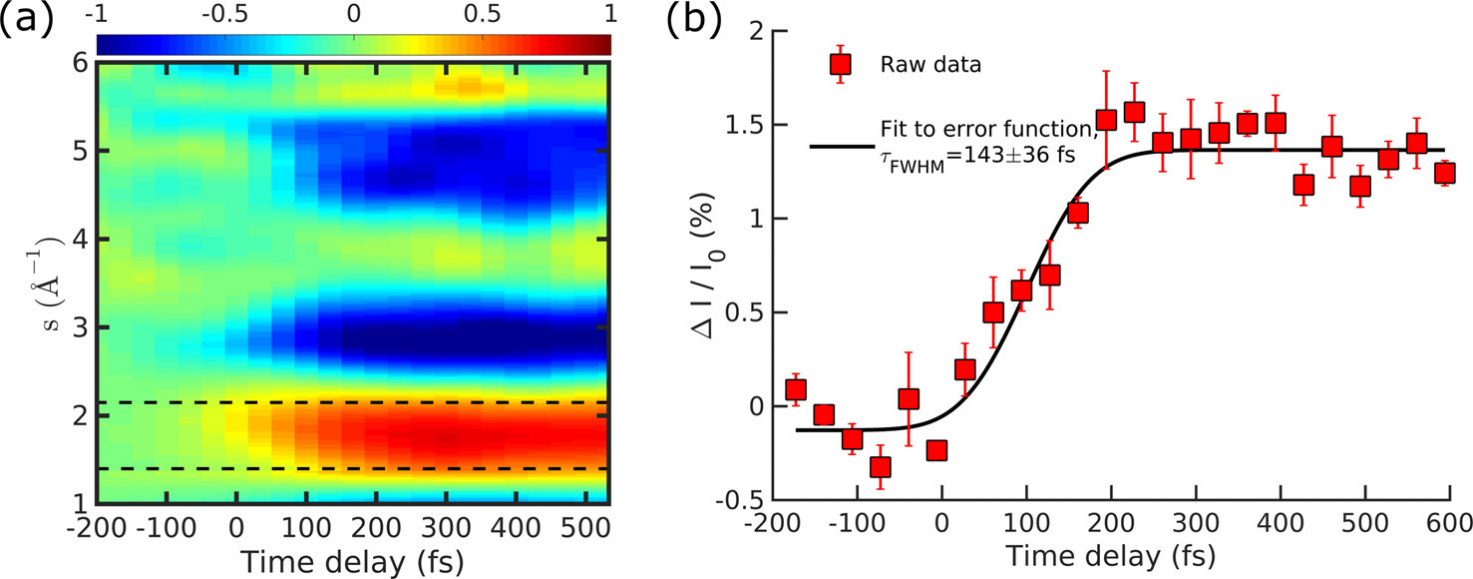
Temporal resolution characterization by the time-resolved ultrafast CF_3_I photodissociation dynamics. (a) The modified scattering intensity as a function of momentum transfer s and pump-probe time delay t. (b) The red squares show the integrated intensities in the region between the two dashed lines (between *s *=* *1.5 Å^−1^ and *s *=* *2.2 Å^−1^) in panel (a), while the black solid curve shows a best fit of error function to the red squares. The FWHM of 143±36 fs gives the upper limit of the temporal resolution.

The temporal resolution of the gas-phase MeV UED apparatus was characterized using the ultrafast photodissociation dynamics of CF_3_I.^51^ Upon absorption of a 266 nm photon, the C-I bond in CF_3_I is broken within 50 fs, resulting in the energetic ejection of a CF_3_^•^ fragment. This ultrafast structural change was captured in a series of diffraction patterns acquired as a function of the pump-probe delay time. Following background subtraction, 2-dimensional diffraction patterns were reduced to 1-dimensional modified scattering intensity curves, through azimuthal averaging and normalization.^48^ Figure 6(a) shows these modified scattering intensity curves as a function of pump-probe delay time t and momentum transfer $s=\left(\frac{4\pi }{\lambda }\right)\;\text{sin}(\frac{\theta }{2})$, where λ is the de Broglie wavelength of the electron and θ is the scattering angle. The onset and bleaching of features are clearly observed at time delays after time-zero, i.e., the delay step in which the pump and probe beam intersect the sample simultaneously. The temporal evolution in intensities integrated over the *s*-range delimited by the two dash lines Fig. 6(a), is shown as red squares in Fig. 6(b). Without devolution of the actual instrumental response and the molecular dynamics involved, fitting this trace to a simple error function gives a FWHM of 143 ± 36 fs, indicating the upper limit of apparatus temporal resolution.

### B. Spatial resolution

**FIG. 7.**
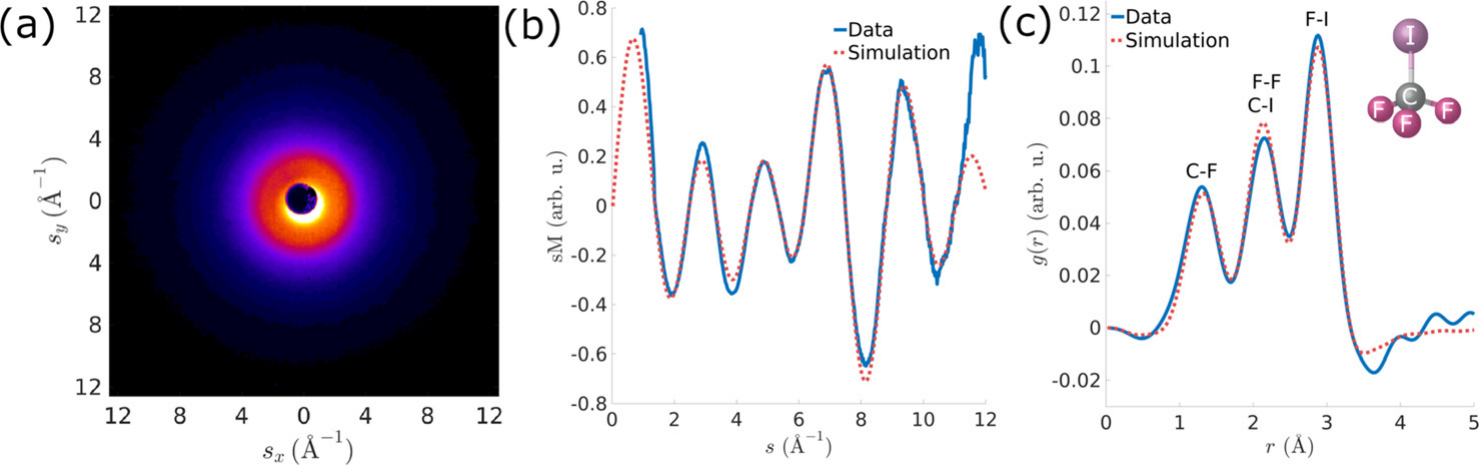
Spatial resolution characterization. (a) Experimental diffraction pattern of CF3I without laser excitation. (b) Modified scattering intensity (blue) extracted from (a) and from simulation (red). (c) The corresponding pair distribution functions from experiment (blue) and simulation (red). The inset shows a cartoon of the molecular structure for CF3I without laser excitation. The peaks corresponding to different bond lengths are labeled.

The spatial resolution in UGED is defined as the finest feature resolved from diffraction data, which can be expressed as *δ* = 2π*/s*_max_, where *s*_max_ is the maximum momentum transfer resolved in the diffraction pattern. Since the gas electron diffraction cross section decreases rapidly with momentum transfer *s*, the spatial resolution is limited by the signal-to-noise ratio (SNR) of the experimental data. The experimental and simulated modified scattering intensity curves, $\mathrm{sM}\left(s\right),$ of the ground state CF_3_I (prior laser excitation) are shown in Fig. 7(b), as the solid blue and dotted red curves, respectively. Agreement between experimental and simulated sM(s) up to s = 10 Å^−1^ demonstrated a spatial resolution of 0.63 Å. The experimental and simulated sM(s) curves can be Fourier-transformed into a pair distribution function (PDF) $g\left(r\right)=\int_{0}^{s_{\text{max}}}sM\left(s\right)\text{sin}\left(sr\right)\exp(-ks^{2})ds$, where r is the radial distance in real space. The PDF shows peaks centered at the interatomic distances (or bond lengths) of the molecule, as shown in Fig. 7(c). The spatial resolution δ determines the minimum width of each peaks retrieved in the PDF. However, the interatomic distance (or bond length) can be determined by the peak center in the PDF with an accuracy significantly higher than the spatial resolution, provided that the distances do not overlap within the spatial resolution. The blue solid curve in Fig. 7(c) shows the PDF extracted from Fig. 7(a), which agrees well with simulation result shown by the red dotted curve in Fig. 7(c). The position of the peak corresponding to the C-F bond length is determined as 1.344 ± 0.007 Å,^51^ which agrees with previous measurements from the literature.^56^

### C. Reciprocal-space resolution

**FIG. 8.**
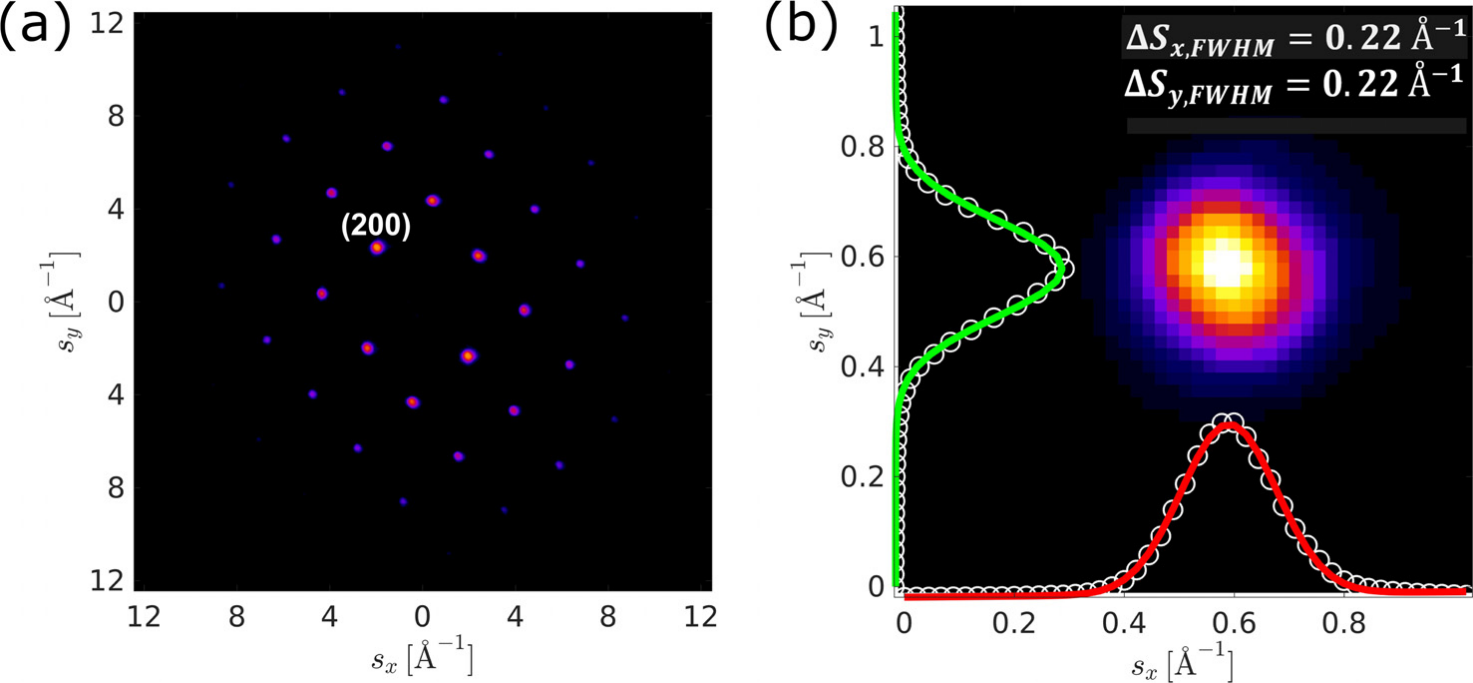
Reciprocal-space resolution characterization. (a) A typical electron diffraction pattern from a single crystal gold sample. (b) A zoom-in view of the (200) Bragg reflection with Gaussian fitted FWHM of 0.22 Å^−1^ as the upper limit of the reciprocal-space resolution.

Reciprocal-space resolution quantifies the extent to which fine features can be unambiguously resolved within a given diffraction pattern. Both the sample property and the electron beam quality contribute to the reciprocal-space resolution. The diffraction pattern of a high-quality single crystal gold sample (shown in Fig. 8) was examined to quantify the upper limit of the instrument reciprocal-space resolution. Using a Gaussian fit to a single (200) Bragg reflection, it was determined to be 0.22 Å^−1^.

## IV. APPARATUS STABILITY CHARACTERIZATION

### A. Electron beam position pointing stability

**FIG. 9.**
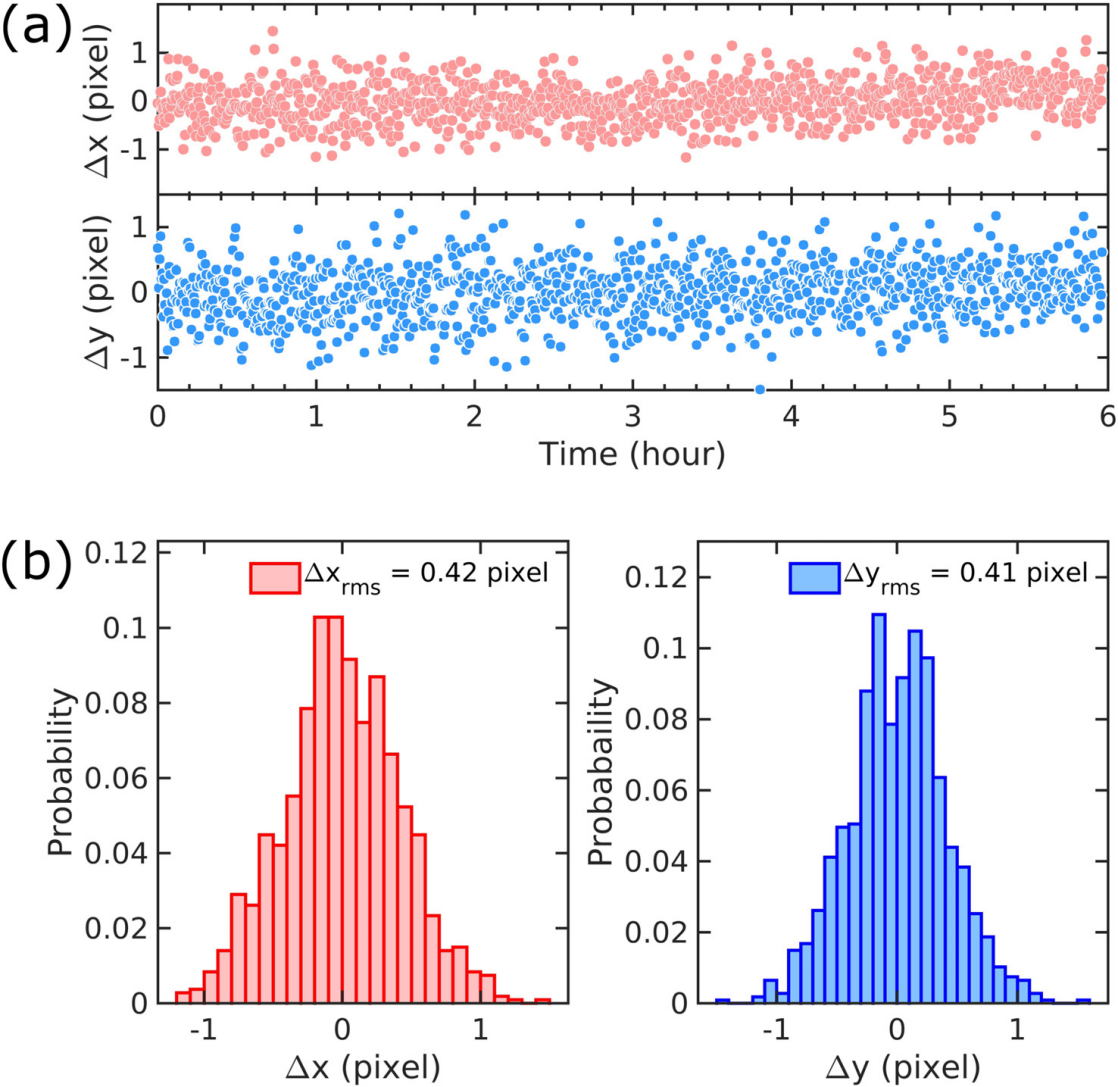
Diffraction pattern centroid jitter in a typical UGED experimental run over 6 h. (a) Time trace of the horizontal (red) and vertical (blue) centroid jitter. (b) Corresponding histograms for the horizontal (red) and vertical (blue) centroid jitter. An rms position pointing jitter of 0.42 pixel corresponding to 14 *μ*m is demonstrated in both directions. The pointing jitter at the interaction points is expected to be better than 14 *μ*m, as it is located 3.2 m upstream of the detector.

The electron beam position pointing jitter of the gas-phase MeV UED apparatus in the interaction region can be estimated from the jitter of the diffraction pattern centroid position at the electron detector. The variation in the horizontal (red) and vertical (blue) centroid position jitter of CF_3_I diffraction patterns acquired over a typical 6-h-long experiment is shown in Fig. 9. The rms centroid jitters in both directions by 0.42 pixel, which corresponds to 14 *μ*m at the detector. This measurement can also be used as an upper limit of the rms electron beam pointing jitter at the interaction point, which is 3.2 m upstream of the detector. Given the micrometer-level pump laser position pointing jitter,^46^ the pump-probe spatial overlap was well maintained within the experiment.

### B. Pump-probe time-zero stability

**FIG. 10.**
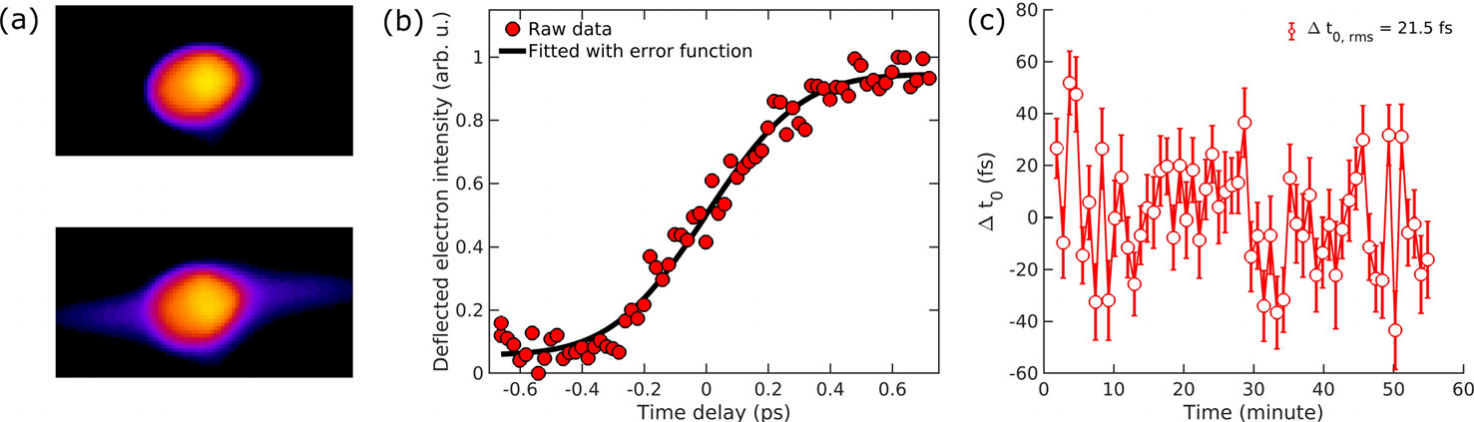
Characterization of pump-probe time-zero stability by the plasma lensing effect. (a) Top graph shows the electron beam profile on the electron detector before time-zero, while the bottom graph shows that at pump-probe time delay *t *=* *600 fs. (b) Intensities of electrons diffracted by the plasma field (red circles) as a function of the pump-probe time delay. An error function (black curve) is fitted to the raw data to determine the onset of the process as a measurement of time-zero. (c) A trace of time-zero changeover one hour monitored by the plasma lensing technique. The error bars reflect the fitting uncertainty in the estimated time zero change.

Long-term time-zero stability is a key to the success gas phase diffraction experiments, given the diffuse nature of the signal and, therefore, long integration times. The pump-probe time-zero of a UGED experiment can be determined using the plasma lensing method.^57,58^ To implement this technique, the intensity of the pump laser is increased to 1.3 × 10^14^ W/cm^2^ by focusing the laser spot size to 50 *μ*m FWHM. The gas molecules are ionized by the pump laser and produce a plasma. In this plasma, electrons with excess kinetic energy diffuse rapidly, while the ions, which are much heavier than the electrons, remain stationary on a picosecond time scale. This results in a net charge redistribution within the plasma and generation of an intense electric field along the direction of the laser polarization. When an electron beam traverses the plasma, a fraction of its charge is deflected by the electric field, causing the streak-like features shown in the beam profiles in the bottom of Fig. 10(a). The plasma lensing effect can be quantified by measuring the intensity of deflected electrons in the electron beam profile as a function of pump-probe time delay, shown in Fig. 10(b). Without devolution of the underlying plasma dynamics and the instrumental response, time-zero can be estimated by fitting this trace to an error function. Given its short acquisition time (1 min), the plasma lensing technique was used to monitor the long-term time-zero stability. As shown in Fig. 10(c), the time-zero stability was demonstrated as 21.5 fs rms over one hour.

## V. CONCLUSION AND OUTLOOK

A gas-phase MeV UED apparatus has been experimentally demonstrated with excellent performance for the study of photoinduced structural dynamics in gas-phase reactions. The gas-phase MeV UED apparatus delivers electron beams at the kinetic energy of 3.7 MeV with 2 fC charge per pulse at a repetition rate of 180 Hz. The successful UED study of the ultrafast dissociation dynamics of CF_3_I demonstrates the machine's unprecedented stability and performance, capturing C-I bond cleavage with 65 fs rms (150 fs FWHM) temporal resolution and 0.22 Å^−1^ and 0.63 Å reciprocal-spatial and spatial resolutions, respectively.

Research and development efforts continue to be devoted to further improving the gas-phase MeV UED performance. For example, a THz-driven electron pulse compression technique has demonstrated a factor of 3 compression in electron bunch length.^59^ The magnitude of compression achieved through this technique could be directly improved by increasing the input THz pulse energy, paving the way toward the delivery of sub-10 fs electron bunches to gas-phase MeV UED experiments. THz streaking has been demonstrated as a direct characterization tool for the time-of-arrival jitter between the electron and laser beams for gas-phase MeV UED experiments.^60^ A project to upgrade the laser and RF system for 1 kHz operation is being carried out, aiming to significantly improve the data acquisition efficiency to achieve higher SNR. Furthermore, a direct electron detector is being developed to enable shot-by-shot readout for implementation of timestamping and data resorting tools to enhance the SNR of the gas diffraction data.^61^
